# Array comparative genomic hybridization analysis discloses chromosome copy number alterations as indicators of patient outcome in lymph node-negative breast cancer

**DOI:** 10.1186/s12885-019-5737-7

**Published:** 2019-05-30

**Authors:** Ryoko Kikuchi-Koike, Kazunori Nagasaka, Hitoshi Tsuda, Yasuyuki Ishii, Masaru Sakamoto, Yoshihiro Kikuchi, Shiho Fukui, Yuko Miyagawa, Haruko Hiraike, Takayuki Kobayashi, Takayuki Kinoshita, Yae Kanai, Tatsuhiro Shibata, Issei Imoto, Johji Inazawa, Osamu Matsubara, Takuya Ayabe

**Affiliations:** 1 Department of Obstetrics and Gynecology, Teikyo University School of Medicine, Tokyo, Japan; 2 Department of Basic Pathology, National Defense Medical College, Saitama, Japan; 3 Division of Molecular Surgical Oncology, Department of Surgical Research, Sasaki Institute, Sasaki Foundation, Tokyo, Japan; 4 Research & Development Management Headquarters, Pharmaceutical & Healthcare Research Laboratories, FUJIFILM Corporation, Kanagawa, Japan; 5 Department of Obstetrics and Gynecology, The Jikei University School of Medicine, Tokyo, Japan; 6 Department of Gynecology, Kyoundo Hospital, Sasaki Foundation, Tokyo, Japan; 7 Department of Gynecology, Ohki Memorial Kikuchi Cancer Clinic for Women, Saitama, Japan; 8 Department of Breast Surgery, National Cancer Center Hospital, Tokyo, Japan; 9 Division of Molecular Pathology, National Cancer Center Research Institute, Tokyo, Japan; 10 Division of Cancer Genomics, National Cancer Center Research Institute, Tokyo, Japan; 11 Department of Human Genetics, Graduate School of Biomedical Sciences, Tokushima University, Tokushima, Japan; 12 Department of Molecular Cytogenetics, Medical Research Institute and Graduate School of Medical and Dental Science, Tokyo Medical and Dental University, Tokyo, Japan

**Keywords:** pN0 invasive breast cancer, Array-CGH, TNFRSF1A, NR2F1, ABCA3

## Abstract

**Background:**

Patients with lymph node metastasis-negative (pN0) invasive breast cancer have favorable outcomes following initial treatment. However, false negatives which occur during routine histologic examination of lymph nodes are reported to underestimate the clinical stage of disease. To identify a high-risk group in pN0 invasive breast cancer, we examined copy number alterations (CNAs) of 800 cancer-related genes.

**Methods:**

Using array-based comparative genomic hybridization (CGH) in 51 pN0 cases (19 relapsed and 32 non-relapsed cases), the positivities of specific gene CNAs in the relapsed and non-relapsed groups were compared. An unsupervised hierarchical cluster analysis was then performed to identify case groups that were correlated with patient outcomes.

**Results:**

The cluster analysis identified three distinct clusters of cases: groups 1, 2, and 3. The major component was triple-negative cases (69%, 9 of 13) in group 1, luminal B-like (57%, 13 of 23) and HER2-overexpressing (26%, 6 of 23) subtypes in group 2, and luminal A-like subtype (60%, 9 of 15) in group 3. Among all 51 cases, those in group 1 showed significantly worse overall survival (OS) than group 2 (*p* = 0.014), and 5q15 loss was correlated with worse OS (*p* = 0.017). Among 19 relapsed cases, both OS and relapse-free survival (RFS) rates were significantly lower in group 1 than in group 2 (*p* = 0.0083 and 0.0018, respectively), and 5q15 loss, 12p13.31 gain, and absence of 16p13.3 gain were significantly correlated with worse OS and RFS (*p* = 0.019 and 0.0027, respectively).

**Conclusions:**

As the target genes in these loci, *NR2F1* (5q15), *TNFRSF1A* (12p13.31), and *ABCA3* (16p13.3) were examined. 5q15 loss, 12p13.31 gain, and absence of 16q13.3 gain were potential indicators of high-risk recurrence and aggressive clinical behavior of pN0 invasive breast cancers.

## Background

Breast cancer is one of the commonest malignancies in women, and mainly occurs in middle-aged or older women with an estimated 1.7million cases and 521,900 deaths in 2012 [1]. For the past decades, the treatment of breast cancer has experienced several changes by tissue-based biomarker and comprehensive analysis of gene expression profiles based on cDNA microarrays has revealed distinct intrinsic subtypes of breast cancer [2–4]. This intrinsic subtype classification was improved [5] and then modified as “surrogate” immunohistochemical subtypes comprising luminal A-like, luminal B-like (HER2-positive and HER2-negative), HER2-overexpressing, and triple-negative subtypes [6]. This immunohistochemical classification is clinically applied for treatment decisions and prediction of patient prognosis [7–9].

Lymph node metastasis-negative (pN0) invasive breast cancer was reported to be associated with good prognosis, with 20–30% 10-year recurrence rate [10–12]. The pN0 breast cancers were classified into subclasses of low and high recurrence risk according to immunohistochemical subtype or histopathological parameters [10–12]. However, immunohistochemical or histopathological parameters are not quantitative, and low inter-observer reproducibility can be problematic [13, 14]. Therefore, the identification of novel quantitative and reproducible prognostic markers of pN0 breast cancers is of major importance.

Breast cancers have been reported to show a number of genomic alterations, including gene copy number and structural alterations [15–19]. Gene copy number alterations (CNAs) and gene expression profiles play important roles in carcinogenetic pathways and can serve as potential biomarkers for prognostication and treatment decisions. It has been utilized extensively for studying the characteristics of breast cancer. Recently, the CNA profiles of *ASB13* and *SGCZ* genes have revealed significant association with survival outcome in young women with breast cancer [20].

In the present study, we examined the CNA profiles of 800 cancer-related genes in 51 pN0 invasive breast cancers using array-based comparative genomic hybridization (aCGH). pN0 invasive breast cancer has been considered to be at low risk of recurrence for more than 5 years after radical surgery. However, it has been reported that the routine examination of regional lymph nodes may be inadequate for the detection of obscure metastases, and that micrometastases were only identified through labor-intensive multiple sectioning and additional immunostaining. Therefore, other potential biomarkers are needed in the decision-making process for treatment. Based on this background, we attempted to identify gene CNAs that were associated with relapse of cancer and patient death, and we were able to identify several gene alterations that may be useful as prognostic biomarkers.

## Methods

### Tumor samples

We analyzed genomic DNAs of the primary breast cancer tissues resected from patients diagnosed with pN0 breast cancer at the National Cancer Center Hospital, Tokyo, between 1990 and 1994 for the aCGH analysis. We listed 20 cases that did and another 40 cases that did not show relapse. In each case, a part of tumor tissue was acetone-fixed immediately after resection at 4 °C overnight, embedded in paraffin, and stored at room temperature. Acetone fixation was employed to preserve high-quality nucleic acids. From hematoxylin-eosin (HE)-stained sections of the tissue blocks, a sufficient amount of genomic DNA was available from 19 relapsed cases and 32 non-relapsed cases.

The mean patient age was 52 years, ranging from 29 to 78. Histological type of cancers consisted of 47 invasive ductal carcinomas, no special type (IDCs-NST); three invasive lobular carcinomas; and one squamous cell carcinoma. The present study was approved by the internal review board for ethical issues of the National Cancer Center and National Defense Medical College. We utilized a strict set of inclusion criteria for patients in the study as follows. Patients were diagnosed with pN0 breast cancer at the National Cancer Center Hospital. All patients provided written informed consent, in accordance with ethical guidelines at the National Cancer Center Hospital. At the time of enrolment in the study, patients had not received adjuvant chemotherapy and tamoxifen was administered to hormone-receptor-positive cases. Each tissue sample was reviewed by a pathologist to confirm the diagnosis and that the sample met inclusion criteria.

### Genomic DNA isolation and labeling

DNA was isolated from 10 sheets of 10-μm-thick acetone-fixed paraffin-embedded tissue sections. Based on examination of HE-stained slides, over 60% of constituent cells in the tissue were confirmed to be tumor cells. These sections were cut with needles or laser microdissection (Leica Microsystems, Tokyo, Japan). Total genomic DNA was isolated with a DNA isolation kit (Gentra Puregene Tissue Kit, Qiagen, Hilden, Germany) following the manufacturer’s instructions. DNA was quantitated using the Nanodrop spectrophotometer. DNA quality was assessed by evaluating the sample’s A260/A280 ratio and its integrity by agarose gel electrophoresis. Reference DNA was derived from a pool of normal female peripheral blood samples. Isolated tumor and reference DNAs were cleaved with *DpnII* and labeled with Cy3- and Cy5-dCTP (GE Healthcare, Tokyo, Japan), respectively, using the random priming method.

### Array-based comparative genomic hybridization (array CGH) using the BAC array

The Bacterial Artificial Chromosome (BAC) array used was constructed in the Fujifilm Advanced Research Laboratories based on the BAC array (MCG Cancer Array-800) that was previously constructed in the Department of Molecular Cytogenetics, Medical Research Institute and School of Biomedical Science, Tokyo Medical and Dental University, Japan [21]. This BAC array, which consists of 800 BACs harboring 800 known cancer-related genes, was intended for use in applying data for cancer-specific CNAs for diagnosis [21]. Hybridizations of the BAC array with tumor or reference genomic DNA was performed as described previously [22]. Hybridized slides were scanned with a GenePix 4000B (Axon Instruments, Union City, CA), and acquired images were analyzed with GenePix Pro 6.0 imaging software (Axon Instruments). Copy number gains and losses were defined as changes in the logarithm to base 2 of the tumor to reference signal intensity ratio (T/R) greater than 0.4 and less than − 0.4, respectively.

### Immunohistochemistry and fluorescence in situ hybridization (FISH) test

Immunohistochemistry (IHC) was performed using the EnVision method with primary antibodies against estrogen receptor (ER; mouse monoclonal clone 1D5, Dako, Glostrup, Denmark), progesterone receptor (PR; mouse monoclonal PgR636, Dako), and HER2 (rabbit polyclonal (HercepTest), Dako). ER and PR were defined as negative when < 1% of tumor cells showed nuclear immunoreaction regardless of the staining intensity [23]. HER2 was defined as negative when the IHC score was 0 or + 1, or when the IHC score was 2+ with negative gene amplification by fluorescence in situ hybridization [24]. According to the immunohistochemical subtypes, luminal A-like subtype was defined as ER- or PR-positive, HER2-negative, and histological grade 1 or 2; luminal B-like subtype was defined as ER- or PR-positive, HER2-negative, and histological grade 3 (luminal B-like, HER2-negative), or ER- or PR-positive and HER2-positive (luminal B-like, HER2-positive); HER2-overexpressing subtype was defined as ER/PR-negative and HER2 positive; and triple-negative was defined as ER/PR negative and HER2-negative [7–9].

### Hierarchical cluster analysis

An unsupervised hierarchical clustering method was applied to analyze genomic aberration similarities across the 51 primary tumor samples using Cluster 3.0 and TreeView software programs. The clustering algorithm was set to complete linkage clustering using an uncentered correlation.

### Statistical analysis

Differences in frequencies of parameters were calculated using the chi-square test with or without Yates’ correction or Fisher’s exact test. The Kolmogorov-Smirnov test was applied to the normality of data distribution. Comparison of non-normally distributed data expressed as medians were calculated using the Mann-Whitney U test. Overall survival (OS) curves and relapse-free survival (RFS) curves were drawn with Kaplan-Meier methods, and differences in curves were analyzed using the log-rank test. *P* < 0.05 for a two sided-test was considered the level of significant difference. Ekuseru-Toukei 2015 (Social Survey Research Information Co., Ltd.) or Exel^R^ statistical software (ystat 2006.xls; Igaku Tosho Shuppan, Tokyo, Japan) was used to analyze the data.

## Results

### Comparison between relapsed and non-relapsed groups

The distribution of patient age, clinical stage, histological type, grade, and immunohistochemical subtype did not differ significantly between relapsed and non-relapsed groups (Table 1).

**Table 1:** Clinicopathological features of 51 lymph node-negative primary invasive breast cancers

Clinicopathological features	No. of cases (%)			*P*
	Total	Relapsed group	Non-relapsed group	
Average age(±SD)	54.5 (±11.3)	55.8 (±10.0)	53.8 (±11.9)	
Stage				
I	9	2 (11)	7 (22)	NS
II	35	14 (73)	21 (65)	
III	7	3 (16)	4 (13)	
Histological type				
Invasive ductal carcinoma	47	16 (84)	31 (97)	NS
Invasive lobular carcinoma	3	3 (16)	0 (0)	
Squamous cell carcinoma	1	0 (0)	1 (3)	
Grade				
1	5	1 (5)	4 (13)	NS
2	14	4 (21)	10 (31)	
3	32	14 (74)	18 (56)	
Immunohistochemical subtype				
Luminal A-like	15	4 (21)	11 (34)	NS
Luminal B-like	18	10 (53)	8 (25)	
HER2 overexpressing	6	0 (0)	6 (19)	
Triple negative	11	5 (26)	6 (19)	
Not identified	1	0 (0)	1 (3)	
All	51	19 (100)	32 (100)	

*NS* not significant, *SD* standard deviation

**Fig. 1:**
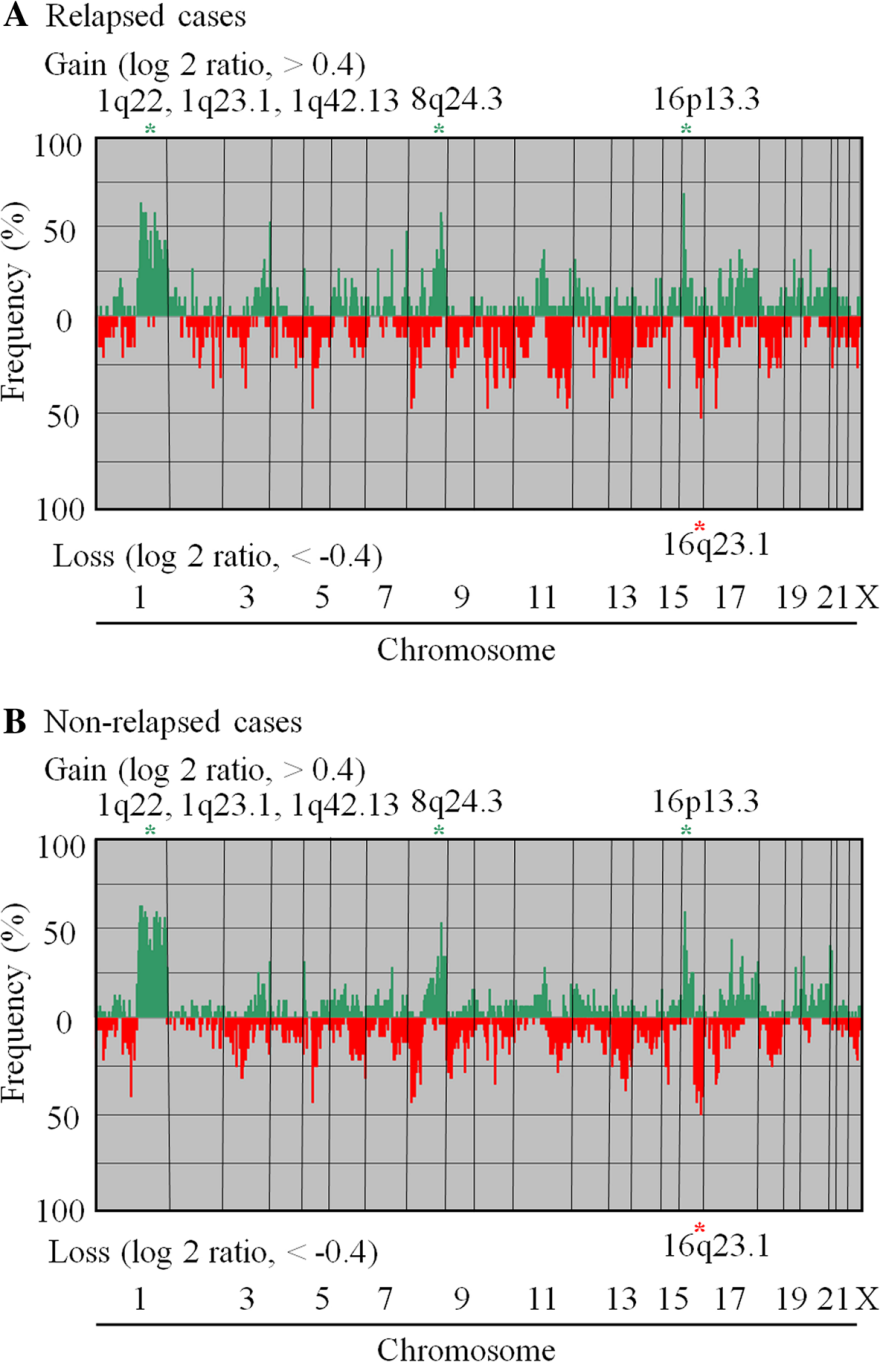
Recurrent genomic abnormalities in (**a**) 19 relapsed and (**b**) 32 non-relapsed lymph node- negative (pN0) invasive breast cancer cases identified based on array CGH. Frequencies of genome copy number gains and losses are plotted as a function of genome location, with chromosome 1p to the left and chromosomes 22, X and Y to the right. Vertical lines indicate frequency of gain or loss. Gene copy-number gains and losses are indicated by red and green, respectively. Green asterisks are the regions that showed frequent gains in over 50% of the cases. Red asterisks are the regions that showed frequent losses in over 50% of these tumors. The chromosome loci that frequently showed gains or losses were common between the relapsed group and the non-relapsed group

In the array CGH analysis using an MCG cancer array-800, frequent copy number gains above 50% were detected in the loci of 1q22, 1q23.1, 1q42.13, 8q24.3 and 16p13.3, and frequent copy number losses above 50% were detected in the locus of 16q23.1 of the 51 pN0 breast cancers. Loci that showed frequent CNAs did not differ between the relapsed and non-relapsed groups (Fig. 1, Table 2).

**Table 2:** Frequent gains and losses in chromosomal loci detected in relapsed and non-relapsed groups

	No. of cases (%)			*P*
Loci	Genes	Relapsed group	Non-relapsed group	
A. Gain				
1q21.3	*MLLT11*	8 (42)	17 (53)	NS
1q22	*MUC1*	12 (63)	20 (63)	NS
	*ARHGEF2*	11 (58)	20 (63)	NS
	*PMF1*	10 (53)	17 (53)	NS
1q23.1	*HAPLN2*	11 (58)	18 (56)	NS
	*PRCC*	11 (58)	19 (60)	NS
	*NTRK1*	8 (42)	18 (56)	NS
1q32.1	*PTPN7*	11 (58)	18 (56)	NS
	*RBBP5*	9 (47)	19 (60)	NS
	*PCTK3*	8 (42)	17 (53)	NS
1q32.3	*ATF3*	7 (37)	18 (56)	NS
1q42.11	*TP53BP2*	8 (42)	16 (50)	NS
1q42.13	*ABCB10*	8 (42)	18 (56)	NS
1q44	*AKT3*	7 (37)	16 (50)	NS
3q29	*MUC4*	10 (53)	10 (31)	NS
8q24.3	*BAI1*	11 (58)	17 (53)	NS
	*PSCA*	10 (53)	11 (34)	NS
16p13.3	*ABCA3*	13 (68)	19 (60)	NS
B. Loss				
16q23.1	*MAF*	10 (53)	16 (50)	NS

*NS* not significant, *SD* standard deviation

The average total number of CNAs in the relapsed group was 129, ranging between 23 and 339, with a standard deviation (SD) of 90. The average total number of CNAs in the non-relapsed group was 114, ranging between 3 and 341, with a SD of 81. These averages were not significantly different between these two groups.

### Classification of lymph node-negative primary breast cancers based on unsupervised hierarchical cluster analysis

**Fig. 2:**
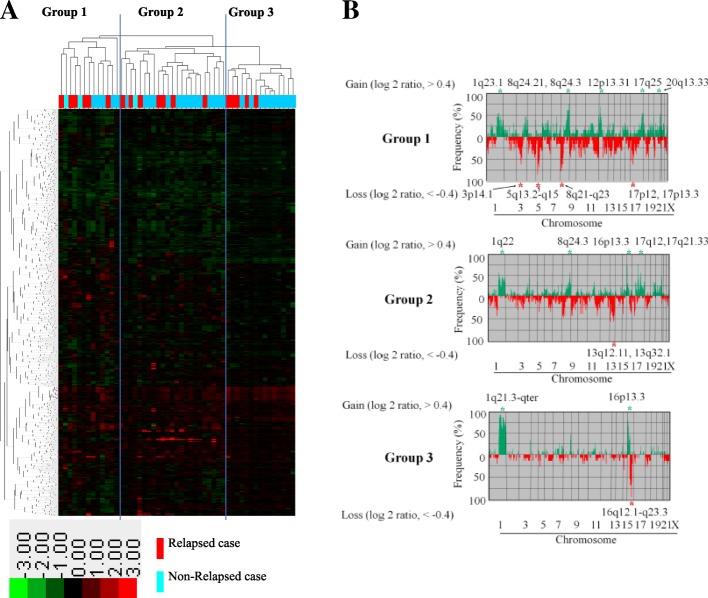
**a** Unsupervised hierarchical cluster analysis of genome copy number profiles measured for 51 pN0 breast cancers. In the horizontal direction, tumor samples are arranged; non-relapsed cases are indicated in light blue, and relapsed cases in red. In the vertical direction, the genes to which the gene copy number was assigned were examined, arranged, and largely classified into two clusters. Gene copy-number gains and losses are indicated by red and green, respectively. A total of 51 tumor samples were classified into three major clusters: groups 1, 2, and 3. **b** Significant differences in genome copy number alteration patterns among groups 1, 2, and 3. In group 1, gains in 1q23.1, 8q24.21, 8q24.3, 12p13.31, 17q25, and 20q13.33 and losses in 3q14.1, 5q13.2-q15, 8p21-q23, 17p12, and 17p13.3 were common. In group 2, gains in 1q22, 8q24.3, 16p13.3, 17q12, and 17q21.33 and losses in 13q12.11 and 13q32.1 were common. In group 3, gains in 1q21.3-qter and 16p13.3 and losses in 16q12.1-q23.3 were common

Unsupervised hierarchical cluster analysis including all 51 tumor samples identified three distinct groups according to the CNA pattern of two clusters of genes in the vertical direction (Fig. 2a). Each cluster consisted of genes at the same loci; genes at the loci on chromosomes 4q, 5q, 6q, 9p, 16q, 18p, and Xp belonged only to the first cluster of genes, and genes at the loci on chromosomes 1q and 16p belonged only to the second cluster of genes. Among these clusters, group 1 had the largest number of CNAs, with an average of 194 (SD 101), ranging from 30 to 341. Group 2 had an intermediate number of CNAs, with an average of 113 (SD 64), ranging from 18 to 249. Group 3 had the smallest number of CNAs, with an average of 65 (SD 40), ranging from 4 to 169 (Fig. 2b). The average number of CNAs in group 1 was higher than that in groups 2 (*p* = 0.026) and 3 (*p* = 0.00036).

Group 1 was characterized by gains of 1q23.1, 8q24.21, 8q24.3, 12p13.31, 17q25, and 20q13.33 and losses of 3p14.1, 5q13.2-q15, 8p21-p23, 17p12, and 17p13.3 (Fig. 2b). This group was mostly (69%, 9 of 13) composed of triple-negative cases, and no case was a HER2-overexpressing subtype (Table 3).

**Table 3:** Immunohistochemical ‘intrinsic’ subtypes of groups 1, 2, and 3

Parameter	No. of cases (%)				*P*
	Total	Group 1	Group 2	Group 3	
Average age (±SD)	54.5 (±11.3)	51.9 (±6.0)	55.0 (±13.4)	56.1 (±10.9)	
Stage					
I	9	3 (23)	3 (13)	3 (20)	NS
II	35	8 (62)	16	(70)	11 (73)
III	7	2 (15)	4	(17)	1 (7)
Histological type					
Invasive ductal	47	12 (92)	23 (100)	12 (80)	NS
Invasive lobular3	3	0 (0)	0 (0)	3 (20)	
Squamous cell	1	1 (8)	0 (0)	0 (0)	
Grade					
1 and 2	19	2 (15)	7 (30)	10 (67)	NS
3	32	11 (85)	16 (70)	5 (33)	
Immunohistochemical subtype					
Triple negative	11	9 (69)	0 (0)	2 (13)	0.000061
Luminal A-like	15	2 (15)	4 (17)	9 (60)	
Luminal B-like	18	2 (15)	13 (57)	3 (20)	
HER2 overexpressing	6	0 (0)	6 (26)	0 (0)	
Not identified	1	0 (0)	0 (0)	1 (7)	
Total	51	13 (100)	23 (100)	15 (100)	

*NS* not significant

Group 2 was characterized by gains of 1q22, 8q24.3, 16p13.3, 17q12, and 17q21.33 and losses of 13q12.11 and 13q32.1 (Fig. 2b). This group was mostly composed of luminal-like subtypes (74%, 17 of 23), four of which were luminal A-like and 13 were luminal-B-like subtype. Six cases (26%) were HER2-overexpressing subtype (Table 3).

Group 3 was characterized by gains of 1q21.3-qter and 16p13.3 and loss of 16q12.1-q23.3 (Fig. 2b). This group was mostly composed of (60%, 9 of 15) luminal A-like subtype (Table 3).

Cases of histological grade 3 were more frequent in groups 1 and 2, than in group 3 (85 and 15%, respectively), and cases of histological grade 1 or 2 were more frequent in group 3 than in groups 1 and 2 (67 and 33%, respectively; *p* = 0.013).

**Fig. 3:**
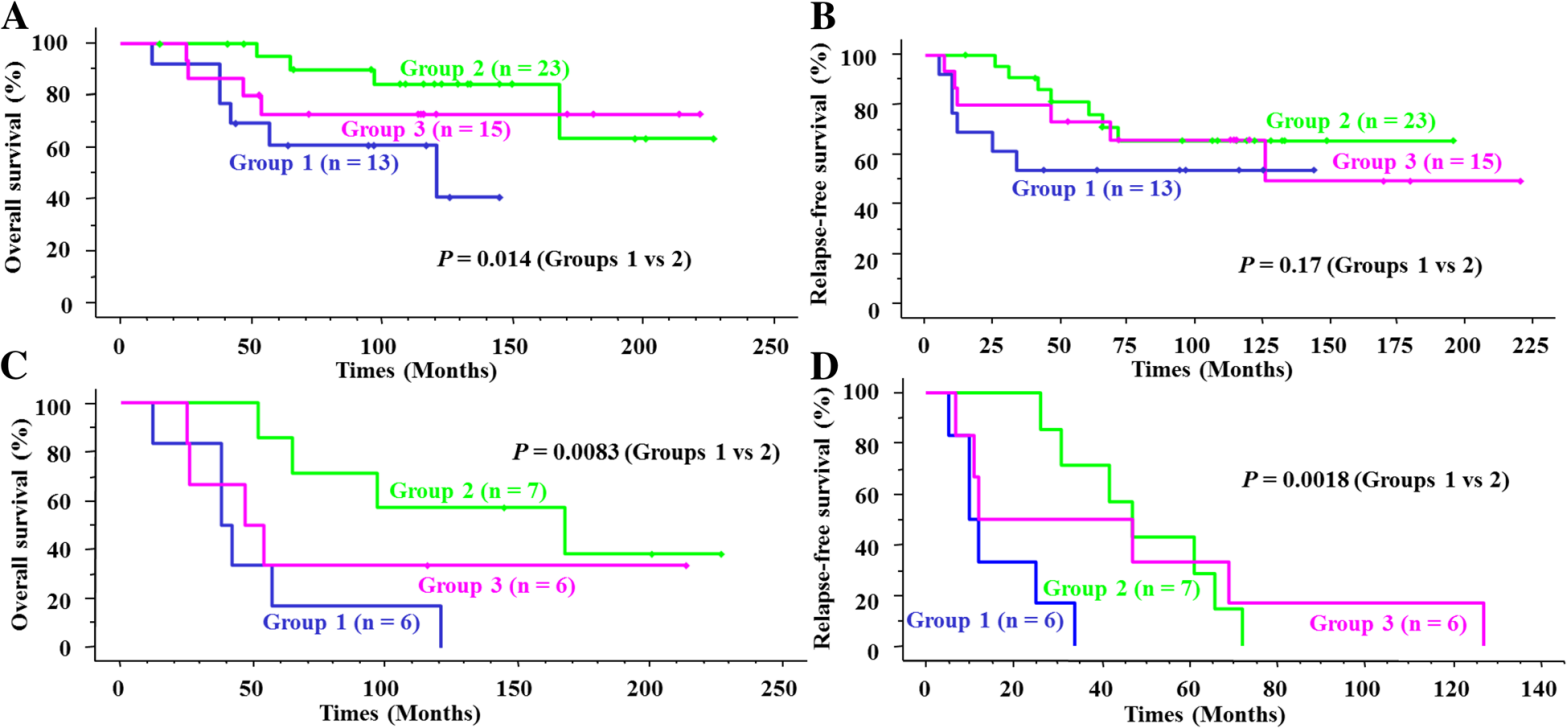
Kaplan-Meier survival curves for the three clusters. (**a** and **b**) Overall survival (OS) (**a**) and relapse-free survival (RFS) (**b**) curves for all 51 pN0 breast cancers. Group 1 shows significantly worse OS than does group 2 (*p* = 0.014). **c** and **d** OS (**c**) and RFS (**d**) curves for 19 relapsed pN0 breast cancers. Group 1 shows significantly lower OS and RFS rates than group 2 (*p* = 0.0083 and 0.0018, respectively)

Among all 51 pN0 breast cancers, the OS rate of group 1 was lower than that of group 2 (*p* = 0.014) (Fig. 3a), whereas the RFS rate was not (Fig. 3b). In contrast, among the 19 relapsed patients, both the OS and RFS rates of group 1 were lower than those of group 2 (*p* = 0.0083 and 0.0018, respectively) (Fig. 3c and d).

### Specific copy number alterations correlated with patient outcomes

**Fig. 4:**
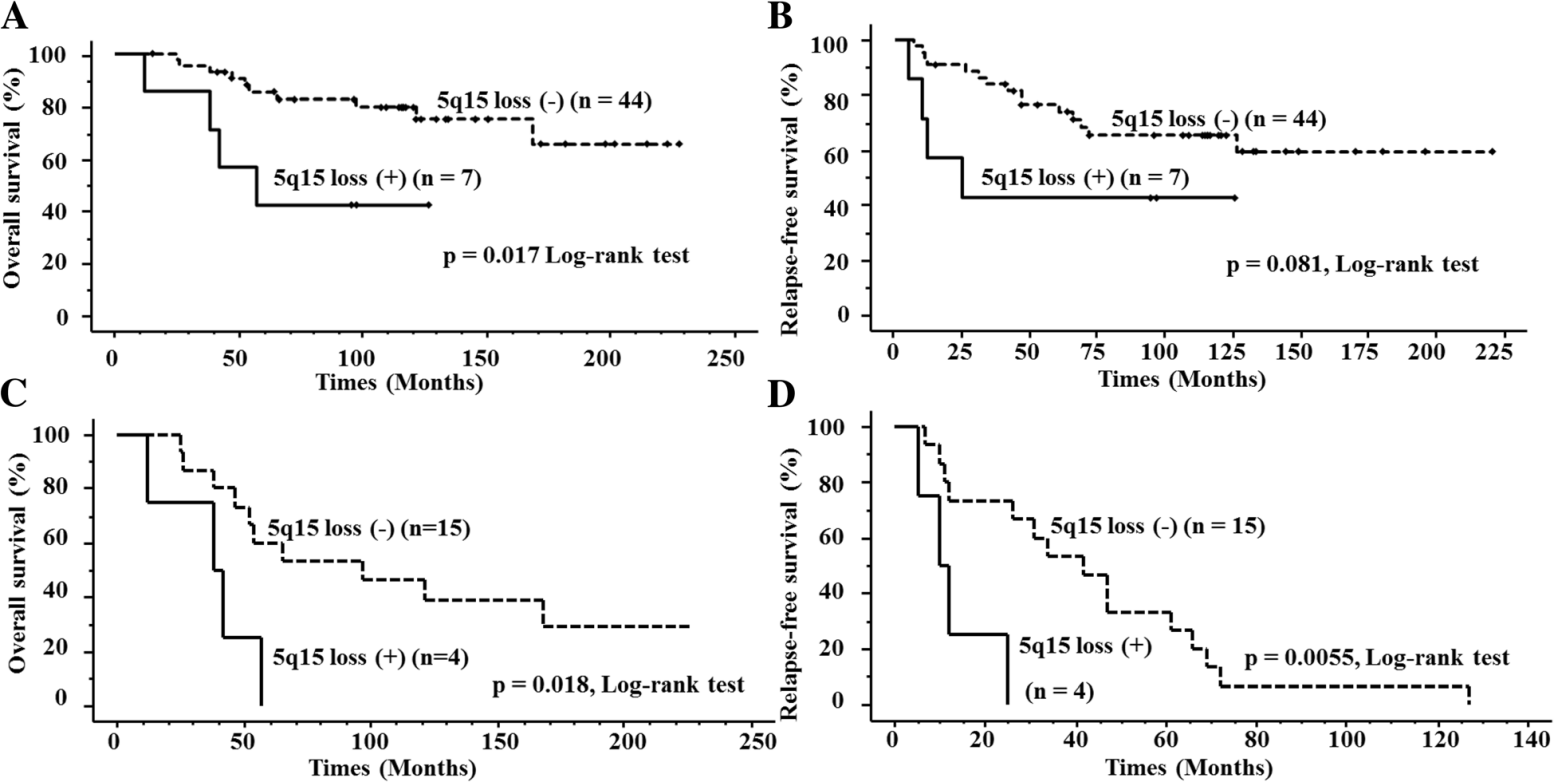
Kaplan-Meier overall survival (OS) (**a**) and relapse-free survival (RFS) (**b**) curves for 51 patients with pN0 breast cancer stratified by 5q15 status. Kaplan-Meier OS (**c**) and RFS (**d**) curves for 19 relapsed patients with pN0 breast cancer stratified by 5q15 status

Among frequent CNAs occurring in groups 1, 2, and 3 shown in Table 4, loss of 5q15 loci was detected only in group 1 tumors (54%, 7 of 13). Cases with the 5q15 loss showed a significantly lower OS rate (*p* = 0.017) and a lower RFS rate (*p* = 0.081) than did those without (Fig. 4a and b). Interestingly, among the 19 patients who suffered relapse, 5q15 loss was significantly correlated with lower OS (*p* = 0.018) and RFS (*p* = 0.0055) (Fig. 4c and d).

**Table 4:**
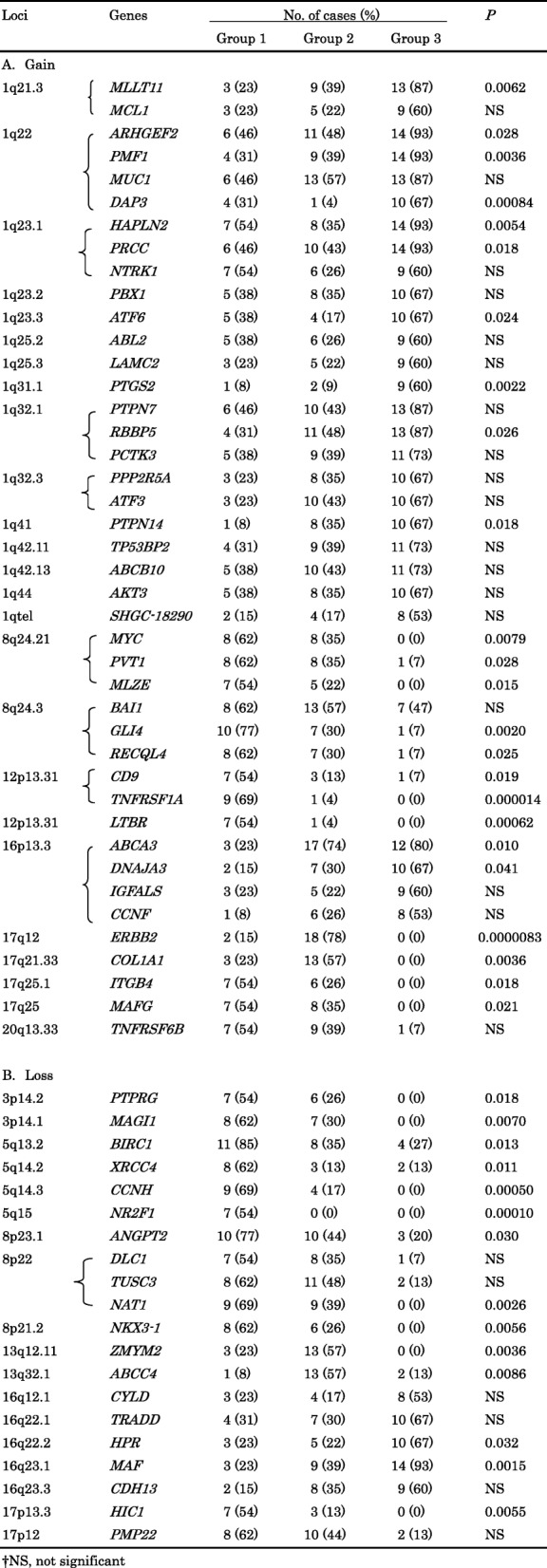
Frequent gains and losses in chromosome loci in groups 1, 2, and 3 identified using hierarchical cluster analysis.

**Fig. 5:**
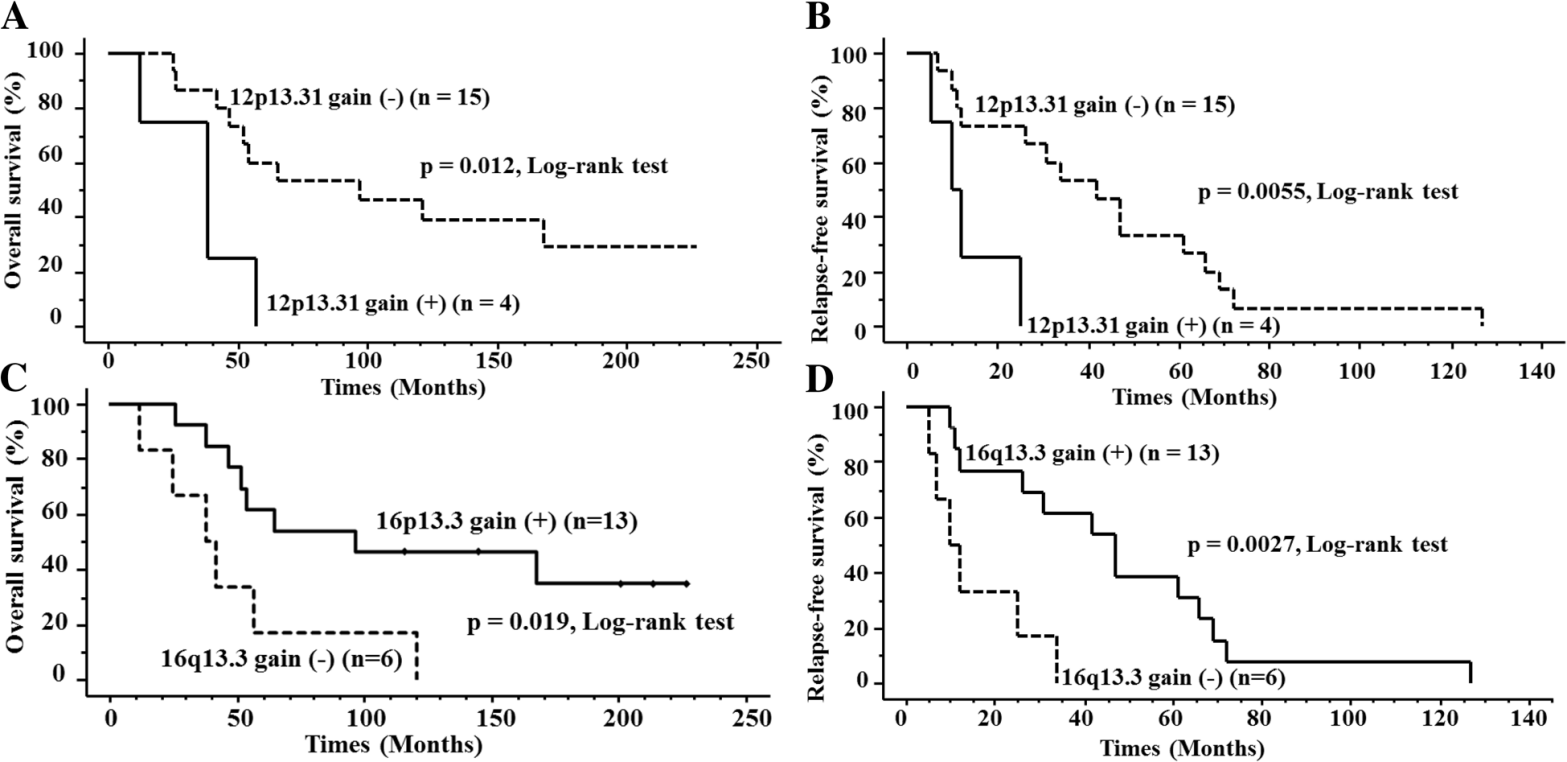
Kaplan-Meier overall survival (OS) (**a**) and relapse-free survival (RFS) (**b**) curves for 19 relapsed pN0 breast cancers stratified based on 12p13.31 status. The OS and RFS curves differed significantly between the groups with and those without gain of 12p13.31. Kaplan-Meier OS (**c**) and RFS (**d**) curves for 19 relapsed pN0 breast cancers stratified by 16p13.3 status. OS and RFS curves differed significantly between the groups with and those without 16p13.3 gain

The gain of 12p13.31 locus was most commonly detected in group 1 tumors (69%, 9 of 13), but was detected in only 1 case among group 2 tumors (4%), and in none of the group 3 tumors (0%). Of the 51 cases, there was no significant difference in OS or RFS curves between the subgroups with and without 12p13.31 gain (*p* = 0.21 and *p* = 0.60, respectively). However, among the 19 patients who suffered relapse, both OS and RFS rates of the cases with 12p13.31 gain were significantly lower than those of the cases without (*p* = 0.012 and 0.0055, respectively) (Fig. 5a and b).

The copy number gain of 16p13.3 locus was common in group 2 (74%, 17 of 23) and group 3 (80%, 12 of 15), but was less common in group 1 tumors (23%, 3 of 13). 16p13.3 gain did not affect OS and RFS in any of the 51 cases (*p* = 0.38 and *p* = 0.76, respectively), but among the 19 relapsed cases, both OS and RFS rates were significantly higher with 16p13.3 gain than without (*p* = 0.019 and 0.0027, respectively) (Fig. 5c and d).

## Discussion

Although numerous studies have examined clinicopathological correlation with CNA status [15–19], limited studies have explored the prognostic implication of CNA status in pN0 breast cancer. For instance, the Molecular Taxonomy of Breast Cancer International Consortium (METABRIC) cohort data has demonstrated integrative cluster associations with histopathological subtypes, tumor grades, and lymphocyte distributions [25]. However, as no information is available on well characterized clinical data with long term follow-up in the study, they did not directly explore the prognostic implication of CNA status in pN0 breast cancer. In our study, based on the total number of CNAs and on CNAs of specific chromosome loci using array CGH technology, we attempted to identify high- and low-risk types of pN0 breast cancer. At first, we identified that there were no differences in the total number of CNAs or of CNAs in the specific loci between the relapsed and non-relapsed group. Therefore, we performed an unsupervised hierarchical cluster analysis based on the array CGH dataset from all 51 cases examined.

Based on this cluster analysis, these 51 cases were classified into three clusters, groups 1 to 3, which corresponded to immunohisochemical subtypes. Group 1 was mostly composed of cases belonging to the triple-negative subtype, group 2 of luminal B-like and HER2-overexpressing subtypes, and group 3 of luminal-A like cases.

Such strong correspondence between CNA pattern and “intrinsic subtype” classification was shown in previous reports [15, 26, 27]. Hicks et al. proposed three patterns of genome rearrangements in breast cancer, i.e., sawtooth, firestorm, and simplex. They suggested that cases of the firestorm pattern were frequently Grade 2 or 3 and were accompanied by CNAs of chromosomes 6, 8, 11, 17, and 20, and especially by amplifications of *CCND1* in 11q and of *ERBB2 (HER2)* in 17q [26]. In the present study, group 2 was mostly composed of luminal B-like and HER2-overexpressing subtypes, and frequently carried 1q, 8q, 16p, 17q gains, which was similar to the firestorm CNA pattern [27]. Hicks et al. showed that the simplex pattern was frequent in grade 1 tumors and was characterized by 16q deletion and 16p duplication, often coupled with 8p loss and duplications of 1q and 8q [26]. These characteristics of the simplex pattern were very similar with those of the present group 3, characterized by luminal A-like subtype, gains of 1q and 16p, and loss of 16q.

In contrast, Natrajan et al. demonstrated that the sawtooth pattern was characterized by high histological grade and basal-like subtype, and numerous CNAs were commonly detected [26]. These CNAs included 5q loss and gain of 1q21.1, 8q24.21-q24.23, and 12p13.31, which agreed with the results for group 1 tumors.

Andre et al. classified breast cancer cases into three groups, i.e., non-negative matrix factorization (NMF) classes I to III, according to data of CGH and cluster analysis [15]. A total of 65% of NMF class I cases were triple negative, and 6p gain, 5q loss, and 15q loss were reported to be common. NMF class II included most of the HER2-overexpressing subtype, and was characterized by 17q gain corresponding to HER2 amplification. In NMF class III, 73% were ER-positive, 97% were HER2-negative, and 1q gain and 16q loss were common.

With regard to patient outcome, the OS of group 1 was significantly worse than that of group 2. Unfortunately, we could not identify any significant clinical/biological association with outcome in the non-relapsed groups, but among the relapsed cases, group 1 patients showed shorter OS and RFS than did group 2. Furthermore, we showed that 5q loss, 12p gain, and absence of 16p13.3 gain were correlated with worse patient outcomes in all cases and/or subsets of recurrent cases in pN0 breast cancer patients.

5q15 loss was commonly detected in group 1, and was correlated with shorter OS in all 51 cases and shorter OS and DFS in the 19 relapsed cases. Curtis et al. classified 2000 breast cancer samples into 10 integrated clusters (IntClust 10) composed mainly of a basal-like subtype that frequently exhibited 5q loss, 8q gain, 10p gain, and 12p gain [16]. These characteristic CNAs in the IntCrust 10 were partly compatible with those in the present group 1.

Curtis et al. also indicated that 5q harbored genes encoding numerous signaling molecules or transcription factors, cell division genes, and 5q deletions that can modulate the coordinate transcriptional control of genomic and chromosomal instability and cell cycle regulation within cancer cells [16].

One of target genes at the 5q15 locus was nuclear receptor subfamily 2, Group F, Member 1 (NR2F1) [28]. NR2F1 provokes a reduction in chemokine CXCL12 expression and enhancement of CXCR4 expression, and it stimulates breast cancer cell migration [29]. On the other hand, NR2F1 also functions as a dormancy gene, so suppression of this gene results in growth of ER-positive MCF-7 cells *in vivo* [29, 30]. Based on these observations, enhanced migration and release from the dormant state in tumor cells by the loss of NR2F1 may be associated with earlier relapse [28].

The 12p13.31 gain was selectively detected in group 1, whereas 16p13.3 gain was only detected in groups 2 and 3. The 12p13 gain was shown to be common in the CNA in basal-like subtype [27]. In contrast, 16p gain was shown to be frequent in luminal subtype, and it was correlated with a good prognosis [16].

One of the target genes at the 12p13.31 locus is *TNFRSF1A*. This gene encodes a receptor of tumor necrosis factor-α (TNF-α), and their ligand-receptor interaction is conditional for the presentation of cellular growth, invasion, and metastasis. The development of primary cancers and metastases were inhibited in *TNFRSF1A*-deficient mice [31]. TNFRSF1A expression is reportedly associated with poor prognosis in diffuse large B-cell lymphoma [32]. In addition, an RNA sequence analysis showed that the breast cancer-associated fusion transcript SCNN1A-TNFRSF1A may play a role in the development of breast cancer [33]. Furthermore, Egusquiaguirre et al. have demonstrated that elevated TNFRSF1A levels may predict a subset of breast tumours that are sensitive to STAT3 transcriptional inhibitors [34].

One of the target genes at the 16p13.3 locus is *ABCA3*, which encodes an ATP-binding cassette (ABC) transporter or a family of transmembrane proteins that can transport a wide variety of substrates across biological membranes in an energy-dependent manner [35]. Negative ABCA3 cytoplasmic immunoreaction or decreased ABCA3 expression was significantly associated with lymph node involvement and worse clinical outcome [36].

All relapsed cases showing 5q15 loss or 12p13.31 gain experienced relapses within 25 months after tumor resection. Therefore, 12p13.31 gain and 5q15 loss were considered markers of pN0 breast cancers showing highly aggressive clinical behavior, with most recurrences being triple-negative breast cancer (TNBC).

Limitations of this study include its small scale and its retrospective case-control design. The number of genes mounted on the array was 800. Nonetheless, we were able to show specific CNAs that were correlated with worse or better patient prognosis using array CGH technology in invasive pN0 breast cancers. The data acquired were compatible with the results of stricter studies using current single nucleotide polymorphism (SNP) arrays, and are considered applicable for the identification of high-risk pN0 breast cancer, after the results are confirmed for larger scale cohorts.

## Conclusions

In conclusion, a CGH array analysis of pN0 invasive breast cancers sorted the cases into three distinct clusters according to an unsupervised hierarchical cluster analysis. We were not able to identify CNAs specific to the non-relapsed group that could be applicable for avoiding unnecessary adjuvant chemotherapy. However, we did identify several specific CNAs as prognostic markers, i.e., 5q15 (NR2F1) loss, 12p13.31 (TNFRSF1A) gain, and absence of 16q13.3 gain, in all pN0 cases and in the high-risk group of pN0 cases that showed relapse. These specific CNAs could be potential candidates for prognostic biomarkers in pN0 invasive breast cancer for monitoring occult metastases which cannot be detected by present histologic examination of the lymph nodes.

## Data Availability

The datasets analysed during the current study are available from the corresponding author on reasonable request but restrictions apply to the availability of these data, and so are not publicly available.

## Abbreviations

ABC: ATP-binding cassette
BAC: Bacterial Artificial Chromosome
CGH: comparative genomic hybridization
CNA: copy number alterations
HE: hematoxylin-eosin
IDCs-NST: invasive ductal carcinomas, no special type
IHC: Immunohistochemistry
IntClust 10: 10 integrated clusters
NMF: non-negative matrix factorization
NR2F1: nuclear receptor subfamily 2, Group F, Member 1
OS: overall survival
pN0: Lymph node metastasis-negative
RFS: relapse-free survival
SD: standard deviation
SNP: single nucleotide polymorphism
TNBC: triple-negative breast cancer
TNF-α: tumor necrosis factor-α
